# Dental Hygiene

**Published:** 1856-06

**Authors:** 


### DENTAL HYGIENE.

In making a summary retrospect of dental literature for the past few years, I find a strange lack of able treatises upon dental hygiene. We have reasonably well written articles upon almost all other articles, and innumerable ones, no doubt, but that branch of our practice which treats of the prevention of disease, is almost a dead letter in our literature.

The fact is, we have been so continually discussing dental education, amalgamation, *rhysodontrophy*, or some such a word, operative and mechanical dentistry, that our primary wants have been too much overlooked.

The study and application of hygienic treatment should be the first and constant care of the dentist, the very base of his practice, if success and deserved profit be his object, for it is a requirement which precedes all others, and is always present in every patient who has become intelligent enough to understand his own wants, and the skill and care with which they are treated; but, this great system is particularly required in the practice with young children before the adult teeth have made their appearance, which is too often confined merely to the premature extraction of the deciduous, with corrections of various irregularities, and sometimes the occasional filling of carious teeth, and various other purposes.

Hygiene is the care of present conditions of the body, which produce healthful consequences in the future. therefore requires the application of much scientific knowledge, and the exercise of experienced judgment, made daily applicable in the fulfillment of some great law of our organism. Through this is found the care required by the maternal parent, in the selection of the food which should constitute her diet during the period in which the child is dependent upon her for its nutriment; for, as this is found possessed of the required particles which constitute the different parts of the body, with the smallest amount of useless or waste matter, so will the child most rapidly thrive and be capable, through vigorous health, of throwing off morbific influences, which, under differently constituted systems, would be attracted rather than repelled, and thus through an acquired weakness of constitution, become a prey to most of the maladies prevalent.

We are made most sensible of the truth of this fact in the almost universally decayed condition of the teeth, scarcely has it been our pleasure to encounter more than two sets of healthy adult teeth during our whole practice, and in these cases, I am confident, that the customary neglect to which the teeth are subjected, would inevitably destroy, and that in a short time, by far the greater majority of those coming under observation; one of these individuals in particular, has impressed me forcibly with the truth of this position, he is between 35 and 40 years of age, is constantly exposed to various temperatures of climate, being a traveler,—smokes and chews inordinately, drinks freely and with considerable regularity, is, in fact, most irregular in all his habits, and yet his dentine is entirely free from all disease, perfectly sound and beautiful in every particular. The gums have always represented to me a slight degree of inflammation, yet if so, have no representation in the consequences of such a condition being present; but the teeth themselves are beautiful, perfectly regular, and I have never seen but one case in which the dental circles were as large, or in which the individual teeth were as large as in this case.

Now this can only be explained, by the fact, that such teeth have been formed under the full and undisturbed requirements of nature, each particle of matter has been furnished by the nutrition, just at the moment required, neither being burthened by too much of the animal, or of the earthy, all the tissues entering into the formative processes, have been undisturbed in their functions, therefore performing their duties in the most perfect manner, and of course observing that timely order, which permits the proper anticipating development to take place, so that in such a process, the maxilla presented room in abundance for the teeth to take their places, and in regular order; the bones of the body were similarly developed, forming a secure base and support to those muscles and ligaments, which otherwise distort and derange the true relation; to such perfection morbific agents are innocuous, and a lifetime will pass without degeneracy and decrepitude.

To a superficial glance this may appear drawn too high, that such exceptions do not establish rules, but I contend that it is these exceptional cases which do establish rules of the most universal application, because they prove that in the absence of known abuses, great laws are established, producing, when uninterrupted, admirable perfection; means entirely suitable to the wants are admirably supplied by an infinite wisdom, and the laws of animal economy being derived from this source are universal as are all other natural laws. We, as dentists, have not only to do with these grave formative principles, but to the correcting of and infringement upon, the laws of health affecting the teeth in all its conditions; we, therefore, turn from these cases respecting purely maternal guardianship to those more properly within the sphere of office practice. In the first place, few practitioners recognize the fact, that there is even a remote possibility of preserving, through life, imperfectly formed teeth, by a judicious and timely management of children through and after the period of dental replacement, but it is not to be surprised at, when you find many proposing to practice, entirely ignorant of the age of respective eruptions, as well as the true causes which operate to the ultimate destruction of almost all teeth, certainly they appreciate a few of the causes, but are even scrupulous in absenting many of great importance, how then can such institute a prevention? The first cause operating to produce dental caries is, imperfect formation and premature development, caused by irregularities of diet, regimen, order,

&c., in the maternal parent of which we have briefly spoken, and producing soft and chalky teeth on the one hand, and irregularity and general unfitness of means to an end in the other; a secondary cause, is the defective physical formation in the teeth themselves, inviting and fully assisting, by their irregularity, depression and corrugation, the action of all acrimonious fluids, which of course bathe their surfaces; such morbid influences acting upon connecting soft tissues, transmit them, by direct means, through the mucous membrane and digestive processes to the whole system, thus giving, by a reflex action, another fearful source of diseased teeth. Besides these, there are other causes, as well as many subdivisions of the foregoing, but these will suffice to extend this article, perhaps to the limits of your convenience, and we therefore, will confine our remarks to a brief consideration of the treatment to be adopted in prevention of these evils. During fœtal existence, too much care cannot be had over the food taken by the mother, as well as the influences, both mental and physical, which surround her, as these all, in every degree, operate to the advantage or disadvantage of the fœtus. This fact is too generally acknowledged to require any argument to sustain; therefore, the only difficulty lies in the just discrimination of the most suitable food to be taken at such time, as well as the guarding against those powerful mental impressions to which females are so peculiarly liable, and which sometimes effect the most astonishing revulsions in the whole economy.

Food which is most nutritive and easily digested is that which is much the most preferable, excluding large proportions of meats, high seasoned dishes and rich gravies; free quantities of stimulating drinks, particularly those engendering feverish excitement, disturbed circulation and incomplete aliment, of course forcing matter unprepared into the blood for nutritive distribution; all erratic feeling and passionate excitement, acts, either to the entire suspension of digestion, or the premature elimination of matters which burden the nutritive and formative economy, perhaps more than any one irregularity. It is evident from these few facts that fœtal growth cannot proceed under such a pressure of active derangement, nor can we have those parts entering into the formation of the teeth well prepared for their future labors, when the very germ of their origin is defective and disturbed. Again, at birth, the food of the mother is that from which the infant's life is sustained and its parts formed, the teeth during all this period are being moulded and composed, and the formative particles are derived from the food of the parent, which, if not fully furnished with those matters demanded in the structure of perfect dentine and enamel, their structure must of necessity be actively defective. Anticipating their formative growth and eruption, the health of the infant should be relatively cared for, as regards its cleanliness, exercise, free air, and pure atmosphere. The entire cleanliness of the mouth should at all times have due consideration, both before and after first dentition, at which time ordinary nutritive food should be given with but a very small proportion of meats. The teeth should be carefully brushed twice daily, and the gums kept in perfect health, presenting a hard surface and rosy pink color. The extraction of the temporary teeth will only be found necessary to prevent irregularities in the due relation of the permanent, which must be carefully guarded by securing timely room for the just advent of all the permanent front teeth, either by the extraction of one or more of the back teeth on either side as the case may be, until all stand in their proper places without undue pressure from each other; this can be obtained by skillfully attached ligatures or constantly applied pressure by the patient or by plates, bearing inclined planes, which act in the direction the irregular tooth is required to take. There is nothing more important than to secure to every young patient a dentine free from all pressure of one tooth against another, so that slight spaces may be found between every tooth and its neighbors, the lodgment of all corrosive matters will be absolutely prevented when due cleanliness is had by means of the brush after each meal, as well upon retiring and rising. Any substance that can be introduced between the teeth of comparatively soft fabric will be found advisable in promoting such cleanliness. These few ideas hastily thrown together will only express common facts which every professional man fully understands, but about which few converse or contribute additional facts to our literature. It should be the constant theme of converse with our patients, when courteous politeness will admit its instruction.—*American Journal.*

